# Safety and efficacy of long-term filgotinib treatment in Japanese patients with ulcerative colitis: an interim subgroup analysis of the SELECTION long-term extension study

**DOI:** 10.1093/crocol/otag006

**Published:** 2026-01-28

**Authors:** Katsuyoshi Matsuoka, Erina Hata, Toshihiko Kaise, Christine Rudolph, Toshifumi Hibi

**Affiliations:** Division of Gastroenterology and Hepatology, Department of Internal Medicine, Toho University Sakura Hospital, Chiba, Japan; Medical Affairs, Gilead Sciences K.K., Tokyo, Japan; Medical Affairs, Gilead Sciences K.K., Tokyo, Japan; Global Medical Affairs, Alfasigma GmbH, Munich, Germany; Center for Advanced Inflammatory Bowel Disease, Kitasato University Kitasato Institute Hospital, Tokyo, Japan

**Keywords:** filgotinib, inflammatory bowel diseases, ulcerative colitis

## Abstract

**Background:**

Filgotinib is an oral, once-daily, Janus kinase 1 preferential inhibitor approved for ulcerative colitis. This interim analysis assessed long-term outcomes of filgotinib in Japanese patients with moderate-to-severe ulcerative colitis.

**Methods:**

In the long-term extension of the phase 2b/3 SELECTION trial (SELECTIONLTE; NCT02914535), SELECTION completers (Week 10 responders) continued filgotinib 200 mg (FIL200), filgotinib 100 mg (FIL100), or placebo, and non–responders received FIL200. Here, safety was evaluated over ⁓4 years in Japanese patients who received FIL200 in SELECTIONLTE using exposure-adjusted incidence rates (EAIRs) per 100 censored patient-years of exposure (cPYE). Efficacy was assessed over 96 weeks in Japanese completers and 144 weeks in Japanese non–responders who received filgotinib (FIL200 for completers; FIL100 or FIL200 for non–responders) in SELECTION and FIL200 in SELECTIONLTE by partial Mayo Clinic Score (pMCS), inflammatory biomarkers, and health-related quality of life (HRQoL).

**Results:**

This analysis included 86 patients (15 completers, 37 non–responders). EAIRs for all infections and herpes zoster were 54.4 and 3.9 per 100 cPYE, respectively. Among completers, the pMCS remission rate increased (LTE Week 96: 69%); and HRQoL and biomarker remission rates remained >50% (LTE Week 96: 85% and 54%, respectively). In non–responders, pMCS, HRQoL, and biomarker remission rates increased (LTE Week 144: 69%, 62%-67% and 60%-69%, respectively).

**Conclusions:**

Filgotinib demonstrated an acceptable long-term benefit–risk profile in Japanese patients, maintaining and improving symptomatic and HRQoL remission for up to 3 years. Japanese patients had a numerically higher incidence of infections and herpes zoster than the overall SELECTIONLTE population.

## Graphical Abstract

**Graphical Abstract:**
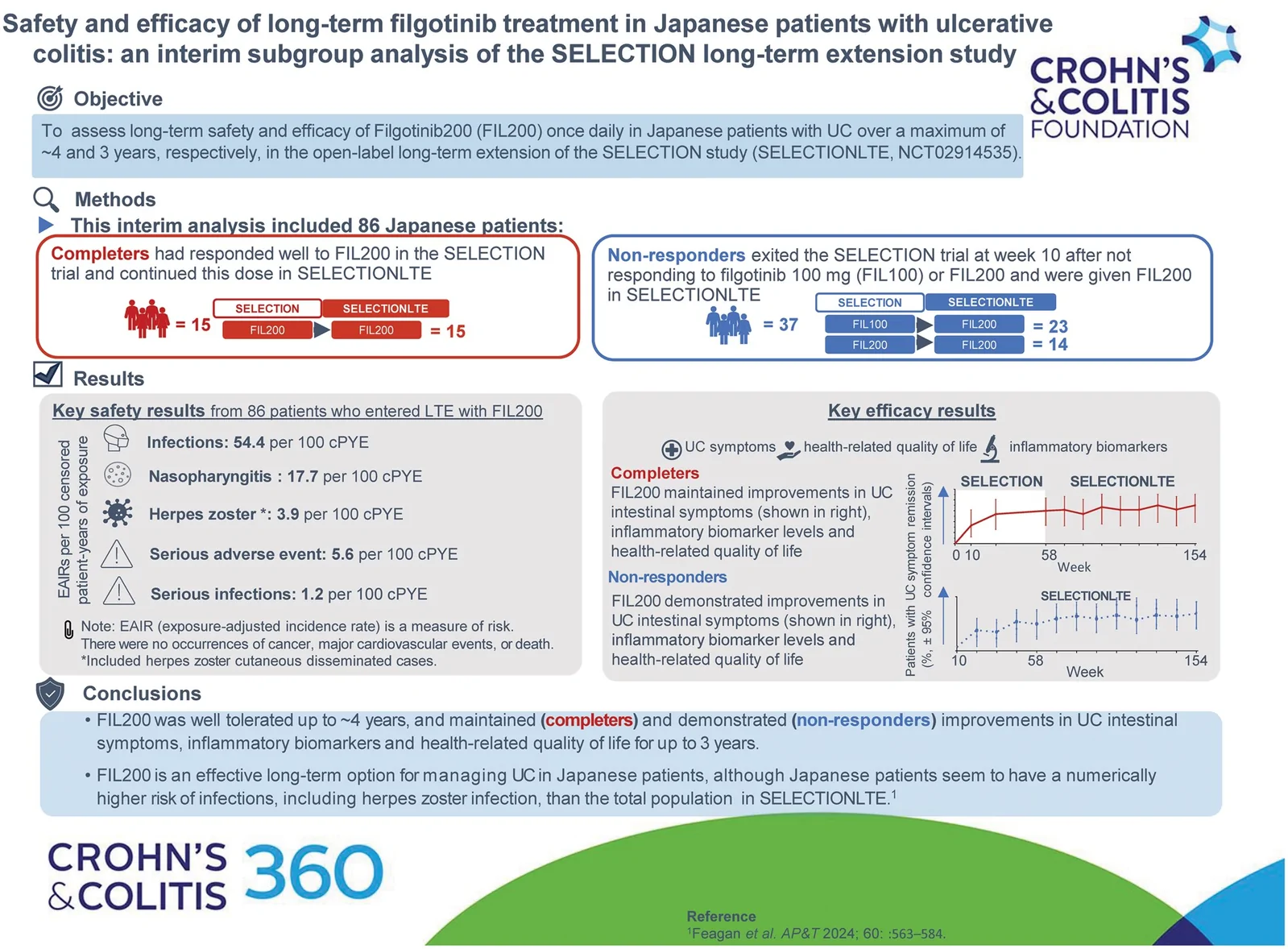

## Introduction

Ulcerative colitis (UC) is a chronic inflammatory bowel disease (IBD) of the colonic mucosa, with symptoms such as bloody diarrhea, urgent bowel movements, fatigue, and abdominal pain.^1^^,^^2^ The incidence of UC in Japan has been increasing since the 1970s, coinciding with the westernization of dietary habits.^3^ From 2010 to 2019, the crude prevalence of UC in Japan increased from 158 to 266 per 100 000 population.^4^

There is currently no curative treatment available for UC. In Japan, treatment goals for UC align with those in many other countries. Treatment goals in Japan include treat-to-target aims of achieving reduced stool frequency, improving diarrhea, achieving an absence of rectal bleeding, and achieving a Mayo endoscopic subscore (MES) of 0 or 1, with an adjunct goal of achieving histological remission.^5^^,^^6^ Several treatment options are available in Japan for patients with moderately to severely active UC, including corticosteroids (CS), 5-aminosalicylate, immunosuppressants, immunomodulators (IM), leukocyte apheresis, biologics, and Janus kinase (JAK) inhibitors.^7^

JAK inhibitors are small molecules that inhibit JAK enzymes (JAK1-3 and tyrosine kinase 2) to reduce cytokine signaling and, therefore, inflammation.^8^ JAK inhibitors have some advantages over biologics, including their oral administration and lack of immunogenicity.^9^^,^^10^ Long-term data for the use of JAK inhibitors and their safety and efficacy are of high interest.

Filgotinib is an oral, once-daily, JAK1 preferential inhibitor that is currently approved for the treatment of moderately to severely active UC in Europe, Japan, Singapore, South Korea, Taiwan, and the United Kingdom.^11-16^ In the phase 2 b/3 SELECTION trial (NCT02914522), filgotinib 200 mg (FIL200) was effective in inducing and maintaining clinical remission, and improving health-related quality of life (HRQoL) over 58 weeks of treatment in patients with moderately to severely active UC.^17-19^ The incidence of treatment-emergent adverse events (TEAEs) in the FIL200 group was similar to that of the placebo group.^17^ A recent evaluation of filgotinib in Japanese patients from the SELECTION trial demonstrated that FIL200 may be a viable treatment option for Japanese patients with UC.^7^^,^^17^

Long-term safety and efficacy of filgotinib in patients with UC are being investigated in the ongoing SELECTION long-term extension study (SELECTIONLTE; NCT02914535). A recent interim analysis of SELECTIONLTE data demonstrated that FIL200 maintained symptomatic remission and improved HRQoL during approximately 4 years of treatment.^20^ Given reports suggesting that Asian populations may experience different adverse event (AE) profiles with JAK inhibitors compared with Western populations,^21^^,^^22^ we have analyzed the long-term impact of filgotinib in Japanese patients enrolled in SELECTIONLTE. Here, we report interim findings on the safety and efficacy in this subgroup, with maximum treatment durations of up to 4 and 3 years, respectively.

## Materials and methods

### Patients, interventions, and study design

The study design of the SELECTION trial (Figure S1) has been reported previously.^17^ Briefly, eligible patients aged 18-75 years with moderately to severely active UC were randomized to receive oral once-daily FIL200, filgotinib 100 mg (FIL100), or placebo (PBO) treatment (2:2:1) for 11 weeks in either induction study A (biologic-naive) or induction study B (biologic-experienced). Patients entered the 47-week maintenance study if they were in clinical remission (defined as a MES of 0 or 1, a rectal bleeding subscore of 0, and a stool frequency subscore of 0 or 1 [with a decrease of at least 1 point from induction baseline]) or had a Mayo Clinic Score (MCS) response (defined as a decrease in MCS of at least 3 points and 30% or more from baseline, with a decrease in rectal bleeding subscore of 1 point or more, or an absolute rectal bleeding subscore of 0 or 1) at Week 10 (responders). In the maintenance study, patients were re-randomized to receive either continued treatment of their assigned filgotinib dosage from the induction study or placebo (2:1).

The study design of SELECTIONLTE (Figure S1) has been previously reported.^20^ Patients eligible for enrollment in SELECTIONLTE included completers (defined as patients from SELECTION who were in clinical remission or who had an MCS response at Week 10 and completed the study to Week 58), non–responders (defined as patients who were discontinued from the SELECTION trial at Week 10 because they were not in clinical remission or did not have an MCS response at Week 10), and patients who experienced protocol-specified disease worsening during the maintenance phase of SELECTION (disease worsening was defined as an increase in partial MCS [pMCS] of ≥ 3 points from the Week 10 value on 2 consecutive visits to achieve a score ≥5 or an increase in pMCS on 2 consecutive visits to achieve a score of 9 if the Week 10 value was > 6). Completers continued double-blinded dosing of their assigned treatment in SELECTION until the unblinding of the SELECTION trial on May 6, 2020; after this date, patients who were receiving filgotinib continued treatment with open-label filgotinib at their assigned doses, and patients who were receiving placebo were discontinued. Non–responders in the induction study and patients who experienced protocol-specified disease worsening in the maintenance study received the maximum dose of open-label filgotinib (ie, as permitted in the country in which they resided, which was 200 mg for this population).

This SELECTIONLTE subgroup interim analysis assessed the safety of up to 202 weeks (3.9 years) and efficacy of up to 154 weeks (3.0 years) of filgotinib therapy in Japanese patients who received at least 1 dose of FIL200. The data cut-off date was February 24, 2022. Not all patients had reached Week 202 of the study by the data cut-off date; however, all patients, excluding those who discontinued, had reached Week 154.

### Outcomes and assessments

#### Safety

Long-term safety was the primary endpoint of SELECTIONLTE, which was assessed at long-term extension (LTE) Weeks 2, 4, and 12, and then every 12 weeks for the duration of SELECTIONLTE. Safety was assessed through the occurrence of AEs, and evaluations of vital signs and clinical laboratory tests.

AEs were coded using the Medical Dictionary for Regulatory Activities (version 25.0). TEAEs were defined as AEs that had an onset date on or after the study drug start date and occurred within 30 days after permanent discontinuation of the study drug, and/or AEs that led to the premature discontinuation of the study drug. TEAEs were also summarized by relationship to study drug, severity, and if they led to premature discontinuation of study drug. In general, AEs were considered to be related to the study drug if there was no evidence that the AE had an etiology other than the study drug. TEAEs of interest were all infections, opportunistic infections, serious infections, herpes zoster, gastrointestinal perforations, non–melanoma skin cancer (NMSC), malignancies (excluding NMSC), major adverse cardiovascular events (MACEs), and venous thromboembolic events (VTEs). Serious AEs (SAEs) were defined as events that resulted in death, in-patient hospitalization or prolongation of existing hospitalization, persistent or significant disability or incapacity, a congenital anomaly, or considered to be life-threatening at the time of the event or medically important enough to jeopardize the patient. Exposure-adjusted incidence rates (EAIRs) were calculated as the number of TEAEs per 100 censored patient-years of exposure (cPYE) for TEAEs and TEAEs of interest. cPYE was defined as the exposure in years until the first occurrence of the event or as the total exposure time for patients without an event. Total cPYE to a treatment was calculated as the sum of all cPYE from each patient.

#### Efficacy

*Post hoc* analyses included the proportion of patients in pMCS score and pMCS remission (defined as pMCS of 0 or 1) over time, changes over time for median C-reactive protein (CRP) levels, and the proportion of patients in fecal calprotectin (FCP) remission over time (defined as an FCP concentration of ≤250 µg/g, irrespective of the baseline value). Efficacy was assessed throughout SELECTIONLTE, up to LTE Week 96 for completers and LTE Week 144 for non–responders. Efficacy outcomes for completers (and CRP levels for non–responders) are also reported at selected time points during SELECTION.

##### Health-related quality of life

Changes in HRQoL were exploratory endpoints of the SELECTIONLTE. Disease-specific HRQoL was assessed using the inflammatory bowel disease questionnaire (IBDQ) and by the proportion of patients achieving IBDQ remission (defined as an IBDQ score of ≥170). HRQoL was assessed at selected time points in SELECTION (completers only) and every 48 weeks in SELECTIONLTE.

### Statistical analysis

#### Safety

The SELECTIONLTE safety analysis set included all enrolled patients who received at least 1 dose of study drug. The safety analysis set in this subpopulation analysis comprised all Japanese patients from the SELECTIONLTE safety analysis set who received at least 1 dose of FIL200. Only events that occurred during SELECTIONLTE were included. The 95% confidence intervals (CIs) for EAIRs per 100 cPYE were calculated using the exact Poisson distribution model. Clinical laboratory results are reported as *n* (%).

#### Efficacy

Efficacy was assessed in Japanese completers who received FIL200 in the induction study, maintenance study and LTE (FIL200–FIL200–FIL200) and Japanese non–responders who received FIL100 or FIL200 in the induction study and open-label FIL200 in the LTE (FIL100–FIL200 or FIL200–FIL200). Participants who received FIL200 in SELECTIONLTE but were randomized to the placebo arm in the SELECTION induction or maintenance study, or did not complete the SELECTION maintenance study, were excluded from the efficacy analyses owing to a shorter duration of filgotinib treatment. Continuous variables are reported as mean ± standard error (SE), mean ± standard deviation (SD), or median (range) and are presented as observed. Binary variables (observed and missing data) are presented as n, proportion (%), and 95% CI. Data missing because of patient discontinuation or intermittent missing data were imputed using non–responder imputation (NRI). No missing data imputation was performed if the data were missing because the enrolled patient had not yet reached a given study visit.

The biomarker analysis set included patients from the safety analysis set who had the necessary baseline and on-study measurements to provide results for the specific parameters of interest that could be interpreted. Analyses of biomarkers (FCP and CRP) were conducted in Japanese patients from the biomarker analysis set who received at least 1 dose of FIL200.

### Ethical considerations

Both the SELECTION and the SELECTIONLTE studies were performed in accordance with the International Conference on Harmonisation Good Clinical Practice guidelines and the Declaration of Helsinki. The protocol and amendments were approved by local review boards or ethics committees (Toho University Sakura Hospital Institutional Review Board and others). Informed written consent was obtained from all patients.

## Results

### Participants

Patient disposition for the SELECTIONLTE Japanese subgroup is reported in Table 1. At the data cut-off date, 86 Japanese patients had received at least 1 dose of FIL200 and were included in the safety analysis set. Of these, 52 patients were included in the efficacy analyses: 15 completers who received FIL200–FIL200–FIL200, 14 non–responders who received FIL200–FIL200 and 23 non–responders who received FIL100–FIL200. The remaining 34 patients who received FIL200 in SELECTIONLTE were excluded from the efficacy analyses because they either did not receive filgotinib in the SELECTION induction or maintenance study, or did not complete the SELECTION maintenance study. Of the 86 patients in the safety analysis set, 21 (24.4%) discontinued the study. The most common reason for study discontinuation was the occurrence of an AE (Table 1).

**Table 1:** Disposition of Japanese patients who had received at least 1 dose of FIL200 in SELECTIONLTE.

	FIL200 (*n *= 86)
**LTE safety analysis set,^a^ * n***	86
**Biomarker analysis set,^b^ * n***	80
**Study completion status, *n* (%)^c^**	
** Continuing study**	65 (75.6)
** Prematurely discontinued study**	21 (24.4)
**Reason for premature discontinuation of study, *n* (%)^c^**	
** Adverse event**	13 (15.1)
** Investigator’s discretion**	7 (8.1)
** Withdrew consent**	1 (1.2)
** Current study unblinded and patient confirmed to be receiving placebo**	0
**Study drug completion status, *n* (%)^c^**	
** Continuing study drug**	55 (64.0)
** Completed study drug**	10 (11.6)
** Prematurely discontinued study drug**	21 (24.4)
**Reason for premature discontinuation of the study drug, *n* (%)^c^**	
** Adverse event**	13 (15.1)
** Investigator’s discretion**	6 (7.0)
** Patient’s decision**	2 (2.3)
** Current study unblinded and patient confirmed to be receiving placebo**	0

a The safety analysis set included all enrolled patients who had received at least 1 dose of the study drug.

b The biomarker analysis set included all patients in the safety analysis set who had baseline and on-study measurements that were necessary for interpretable results for specific parameters of interest in this interim analysis.

c Percentages were calculated based on the number of patients in the LTE safety analysis set who received FIL200 treatment.

Abbreviations: FIL200, filgotinib 200 mg; LTE, long-term extension; SELECTIONLTE, SELECTION long-term extension.

As safety data were collected after the SELECTIONLTE baseline, patient demographics and characteristics for the safety analysis set are reported at SELECTIONLTE baseline in Table 2. Efficacy data, however, are presented from SELECTION baseline; therefore, patient demographics and characteristics for completers and non–responders are reported at SELECTION baseline in Table 3. Body weight, disease duration, and total MCS were similar between the completers and the non–responders. Completers had a mean age of 50.7 years. The proportions of patients who used CS therapy was 46.7% for completers (CS only: 40.0%; CS + IM: 6.7%) and 34.4%-40.4% for non–responders (CS only: 13.0%-14.3%; CS + IM: 21.4%-26.1%). The majority of patients had used at least 1 prior biologic agent, and more than half had experienced at least 1 tumor necrosis factor α inhibitor failure.

**Table 2:** Demographics and characteristics at LTE baseline in the safety analysis set.

	FIL200 (*n *= 86)
**Age, mean (SD)**	47 (14.8)
**Female, *n* (%)**	29 (33.7)
**Body weight, kg median (Q1-Q3)**	61.9 (54.8-68.7)
**BMI, kg/m^2^, median (Q1-Q3)**	21.9 (20.7-24.1)
**Partial MCS,^a^ mean (SD)**	4.6 (2.27)
**CRP, mg/L mean (SD)**	4.10 (8.899)
**FCP, μg/g, mean (SD)**	2441 (4584.4)
**Concomitant therapeutic use, *n* (%)**	
** Systemic corticosteroids**	14 (16.3)
** Immunomodulators only**	22 (25.6)
** Systemic corticosteroids and immunomodulators**	12 (14.0)
** 5-ASA**	71 (82.6)
**Prednisone-equivalent dose, mg/day, median (Q1-Q3)**	6.0 (4.0-12.5)

a Partial MCS was defined as the sum of Mayo rectal bleeding, stool frequency, and Physician’s Global Assessment subscores.

Abbreviations: 5-ASA, 5-aminosalicylic acid; BMI, body mass index; CRP, C-reactive protein; FCP, fecal calprotectin; FIL200, filgotinib 200 mg; LTE, long-term extension; MCS, Mayo clinic score; Q1, first quartile; Q3, third quartile; SD, standard deviation.

**Table 3:** Demographics and characteristics at SELECTION baseline for Japanese completers and non-responders in SELECTIONLTE.

	Completers	Non-responders	
	FIL200–FIL200–FIL200 (*n *= 15)	FIL200–FIL200 (*n *= 14)	FIL100–FIL200 (*n *= 23)
**Age, years, mean (SD)**	50.7 (13.5)	45.5 (16.4)	41.5 (14.6)
**Female, *n* (%)**	8 (53.3)	4 (28.6)	4 (17.4)
**Body weight, kg, median (Q1-Q3)**	56.7 (46.6-63.6)	63.7 (61.2-65.2)	62.3 (56.7-71.5)
**BMI, kg/m^2^, median (Q1-Q3)**	21.8 (19.3-22.8)	21.7 (20.7-23.1)	21.6 (19.1-24.1)
**Smoking status, *n* (%)**			
** Current**	1(6.7)	1 (7.1)	1.0 (4.3)
** Former**	7 (46.7)	5 (35.7)	11.0 (47.8)
** Never**	7 (46.7)	8 (57.1)	11.0 (47.8)
**Duration of UC, years, mean (SD)**	8.1 (7.0)	8.9 (7.9)	9.0 (7.6)
**Total MCS,^a^ mean (SD)**	8.5 (1.3)	8.1 (1.2)	8.8 (1.3)
**MES of 3, *n* (%)**	11 (73.3)	12 (85.7)	18 (78.3)
**CRP, mg/L, mean (SD)**	6.9 (14.0)	5.8 (6.1)	6.2 (10.2)
**FCP, μg/g, mean (SD)**	4381.2 (3405.3)	1967.1 (2240.1)	1604.1 (1287.9)
**Concomitant therapeutic use, *n* (%)**			
** Systemic corticosteroids**	6 (40.0)	2 (14.3)	3 (13.0)
** Immunomodulators only**	2 (13.3)	5 (35.7)	5 (21.7)
** Systemic corticosteroids and immunomodulators**	1 (6.7)	3 (21.4)	6 (26.1)
** 5-ASA**	11 (73.3)	14 (100)	22 (95.7)
**Prednisone-equivalent dose, mg/day, median (Q1-Q3)**	5.0 (4.5-13.8)	10.0 (2.5-15.0)	7.5 (5.0-10.0)
**Number of prior biologic agents used, *n* (%)**			
** 0**	6 (40.0)	4 (28.6)	6 (26.1)
** 1**	4 (26.7)	4 (28.6)	9 (39.1)
** 2**	3 (20.0)	4 (28.6)	7 (30.4)
** ≥3**	2 (13.3)	2 (14.3)	1 (4.3)
**Prior biologic agents used, *n* (%)**			
** ≥1 TNFα inhibitor**	9 (60.0)	10 (71.4)	17 (73.9)
** Vedolizumab**	0	2 (14.3)	2 (8.7)
** ≥1 TNFα inhibitorand vedolizumab**	0	2 (14.3)	2 (8.7)
**Prior biologic failure, *n* (%)**			
** ≥1 TNFα inhibitor**	8 (53.3)	9 (64.3)	15 (65.2)
** Vedolizumab**	0	1 (7.1)	0

a MCS was defined as the sum of the endoscopic, stool frequency, rectal bleeding and Physician’s Global Assessment subscores, with the total score ranging from 0 to 12.

Abbreviations: 5-ASA, 5-aminosalicylic acid; BMI, body mass index; CRP, C-reactive protein; FCP, fecal calprotectin; FIL100, filgotinib 100 mg; FIL200, filgotinib 200 mg; MCS, Mayo clinic score; MES, Mayo endoscopic subscore; Q1, first quartile; Q3, third quartile; SD, standard deviation; SELECTIONLTE, SELECTION long-term extension; TNF, tumor necrosis factor alpha; UC, ulcerative colitis.

A plain language summary describing the study population and key outcomes of this study is available in the Supplementary Material.

### Safety

#### Treatment-emergent adverse events

TEAEs and TEAEs of interest for the 86 Japanese patients who had received at least 1 dose of FIL200 are reported in Table 4. The total follow-up for these patients was 240.6 cPYE. Overall, the EAIRs were 228.8 per 100 cPYE for all TEAEs and 5.6 per 100 cPYE for serious TEAEs. The EAIRs for TEAEs of grade 3 or higher and TEAEs leading to study discontinuation were 6.1 per 100 cPYE and 5.9 per 100 cPYE, respectively. There were no deaths reported. The EAIR for infections and infestations was 54.4 per 100 cPYE. The most frequent infection was nasopharyngitis (EAIR of 17.7 per 100 cPYE). A full summary of the infections and infestations is reported in Table S1. There were no occurrences of malignancy (excluding NMSC), NMSC, MACE, or VTE.

**Table 4:** Summary of TEAEs and TEAEs of interest for Japanese patients who had received at least 1 dose of FIL200 in SELECTIONLTE.

	FIL200 (*n *= 86; total cPYE = 240.6)	
	n/cPYE	EAIR per 100 cPYE (95% CI)
**Summary of TEAEs**		
** Overall TEAEs**	84/36.7	228.8 (182.5-283.2)
** Serious TEAEs**	13/234.1	5.6 (3.0-9.5)
** TEAEs grade 3 or higher**	14/230.1	6.1 (3.3-10.2)
** TEAEs leading to premature discontinuation of the study drug**	14/239.2	5.9 (3.2-9.8)
** Deaths**	0/240.6	0.0 (0.0-1.5)
**TEAEs of interest**		
** All infections and infestations**	60/110.2	54.4 (41.5-70.1)
** Opportunistic infections**	2/240.4	0.8 (0.1-3.0)
** Serious infections** ^a^	3/240.3	1.2 (0.3-3.6)
** Herpes zoster**	8/228.1	3.5 (1.5-6.9)
** Herpes zoster cutaneous disseminated**	1/240.5	0.4 (0.0-2.3)
** Gastrointestinal perforations**	0/240.6	0.0 (0.0-1.5)
** Malignancies (excluding NMSC)**	0/240.6	0.0 (0.0-1.5)
** NMSC**	0/240.6	0.0 (0.0-1.5)
** MACEs** ^b^	0/240.6	0.0 (0.0-1.5)
** VTEs** ^b^	0/240.6	0.0 (0.0-1.5)

TEAE severity grades were defined using CTCAE (version 4.03). Patients who discontinued the study drug after the data cut-off date (February 24, 2022) but who were in the database were also included. 95% CIs were calculated using the exact Poisson distribution model.

a Serious infections were: COVID-19 (*n *= 1), diverticulitis (*n *= 1), and pneumonia aspiration (*n *= 1).

b MACE and VTE data were adjudicated by an independent committee in a blinded manner.

Abbreviations: CI, confidence interval; cPYE, censored patient-years of exposure; CTCAE, common terminology criteria for adverse events; COVID-19, coronavirus disease 2019; EAIR, exposure-adjusted incidence rate; FIL200, filgotinib 200 mg; MACE, major adverse cardiovascular event; NMSC, non-melanoma skin cancer; SELECTIONLTE, SELECTION long-term extension; TEAE, treatment-emergent adverse event; VTE, venous thromboembolic event.

The EAIR for herpes zoster/herpes zoster cutaneous disseminated was 3.9 per 100 cPYE. In total, 9 herpes zoster/herpes zoster cutaneous disseminated infections were reported, and all were mild or moderate in severity, with none classified as serious. Narratives for the 9 patients with TEAEs related to herpes zoster infections are reported in Table S2. Of these 9 patients, 1 was reported to have herpes zoster cutaneous disseminated. Four of the 9 cases occurred in the 1st year of SELECTIONLTE. The remaining cases occurred during the second (*n *= 1), third (*n *= 2), and 4th (*n *= 2) years of SELECTIONLTE.

### Efficacy

#### Partial Mayo clinic score

##### pMCS over time

**Figure 1:**
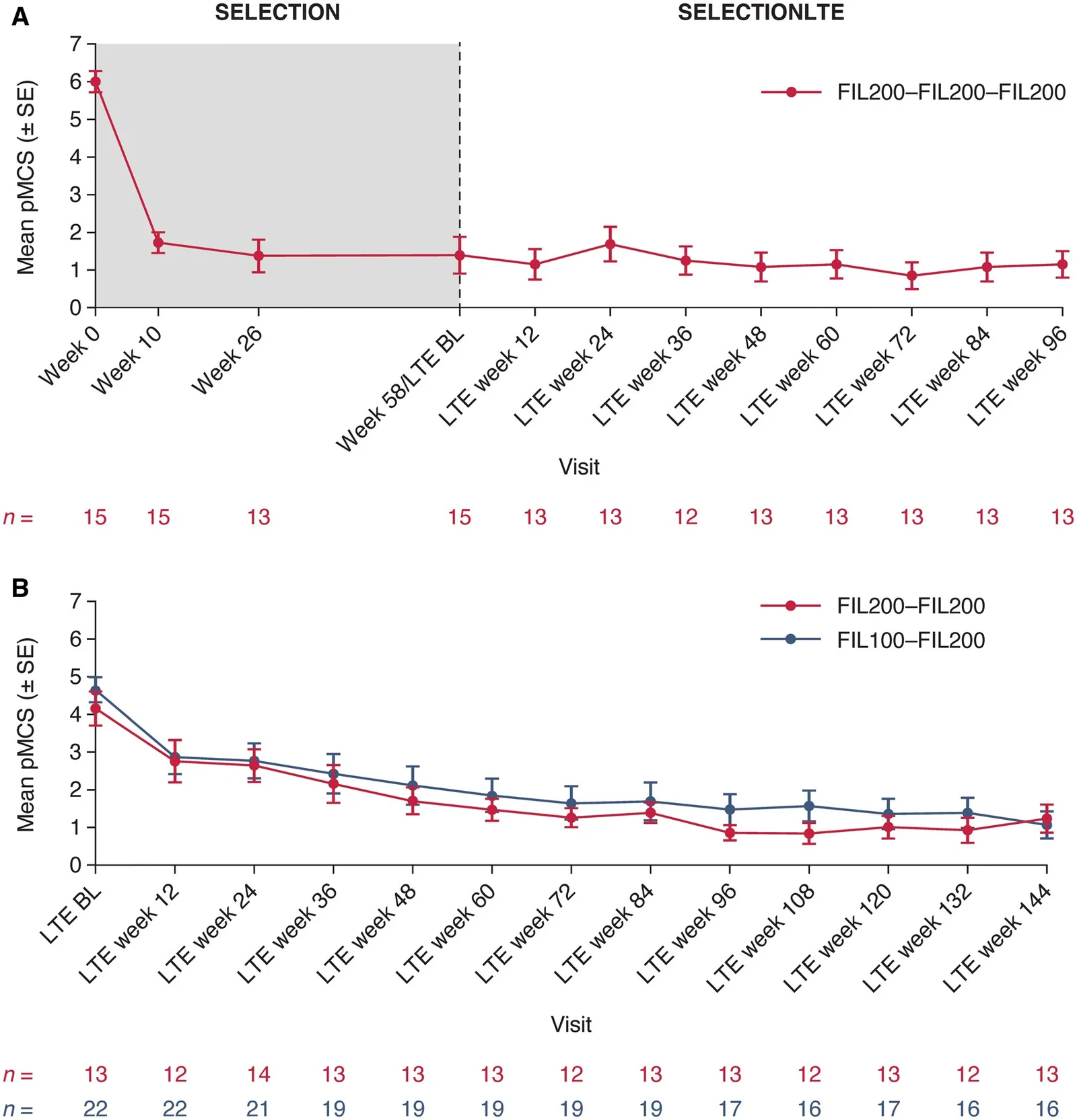
Mean pMCS^a^ over time among Japanese (A) completers and (B) non-responders. No imputation was performed for missing data. ^a^pMCS was defined as the sum of Mayo rectal bleeding, stool frequency, and Physician’s Global Assessment subscores. Abbreviations: BL, baseline; FIL100, filgotinib 100 mg; FIL200, filgotinib 200 mg; LTE, long-term extension; pMCS, partial Mayo clinic score; SE, standard error; SELECTIONLTE, SELECTION long-term extension.

For FIL200–FIL200–FIL200 completers, the mean pMCS at LTE baseline was 1.4 (Figure 1A). The mean pMCS for these patients remained generally stable up to LTE Week 96 (mean pMCS: 1.2) (Figure 1A). For FIL200–FIL200 and FIL100–FIL200 non–responders, the mean pMCS at LTE baseline was 4.2 and 4.6, respectively (Figure 1B). By LTE Week 144, the mean pMCS for FIL200–FIL200 and FIL100–FIL200 non–responders decreased to 1.2 and 1.1, respectively (Figure 1B).

##### pMCS remission in completers

**Figure 2:**
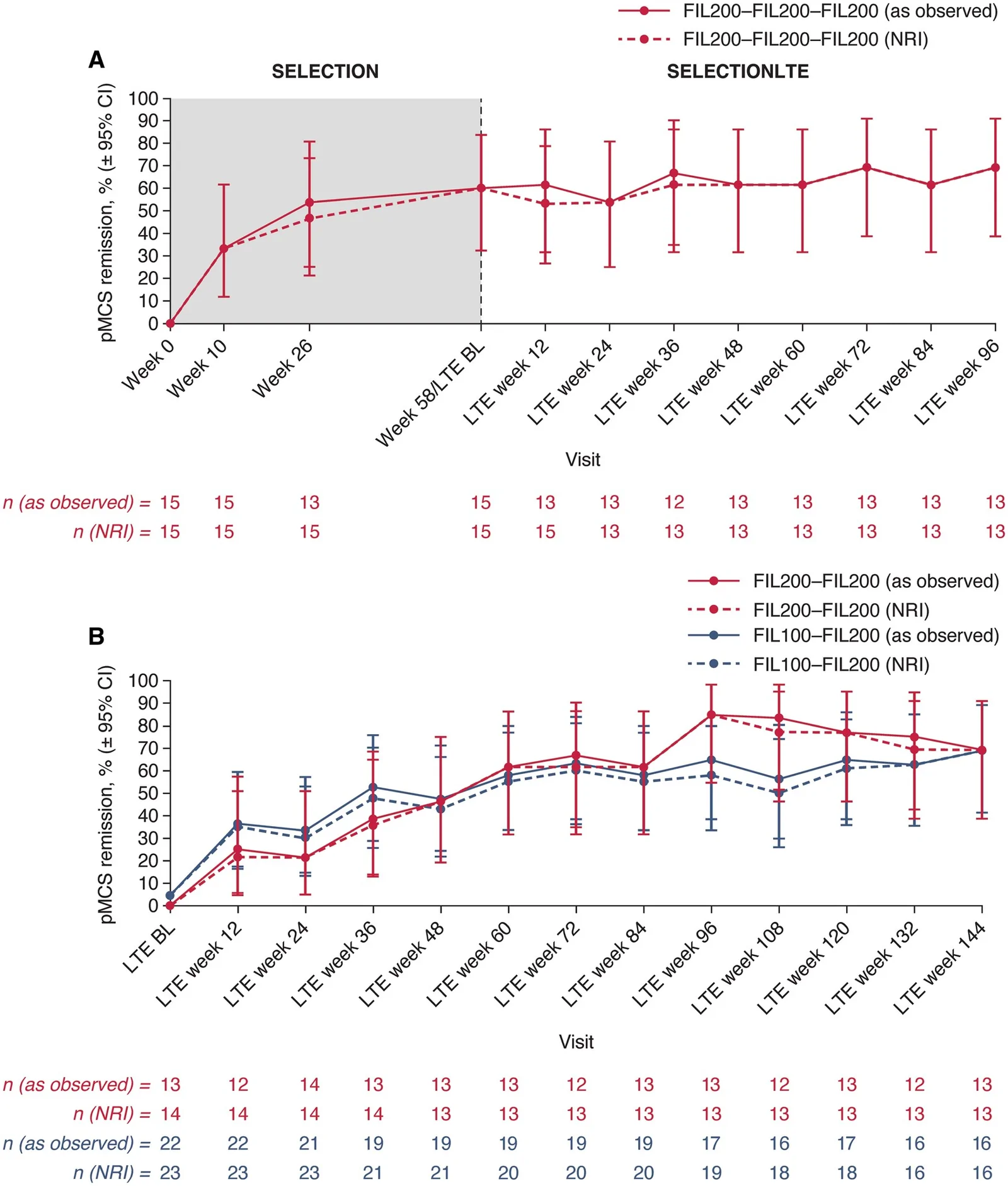
Proportions of Japanese (A) completers and (B) non-responders in pMCS remission^a^ over time. ^a^pMCS remission was defined as a pMCS of ≤1. pMCS was defined as the sum of Mayo rectal bleeding, stool frequency, and Physician’s Global Assessment subscores. Abbreviations: BL, baseline; CI, confidence interval; FIL100, filgotinib 100 mg; FIL200, filgotinib 200 mg; LTE, long-term extension; NRI, non-responder imputation; pMCS, partial Mayo clinic score; SELECTIONLTE, SELECTION long-term extension.

The proportion of patients who were in pMCS remission generally increased over time for completers (Figure 2). In the as-observed analysis, at LTE baseline, 60.0% (9/15 patients) of FIL200–FIL200–FIL200 completers were in pMCS remission; the rate was 69.2% (9/13 patients) at LTE Week 96 (Figure 2A). In the NRI analysis, similar trends were observed (Figure 2A).

##### pMCS remission in non-responders

The proportion of patients who were in pMCS remission generally increased over time for non–responders (Figure 2). In the as-observed analysis, the proportions of FIL200–FIL200 and FIL100–FIL200 non–responders who were in pMCS remission were 0% (0/13 patients) and 4.5% (1/22 patients) at LTE baseline and increased to 69.2% (9/13 patients) and 68.8% (11/16 patients) at LTE Week 144, respectively (Figure 2B). In the NRI analysis, similar trends were observed (Figure 2B).

#### Inflammatory bowel disease questionnaire

##### IBDQ score over time

**Figure 3:**
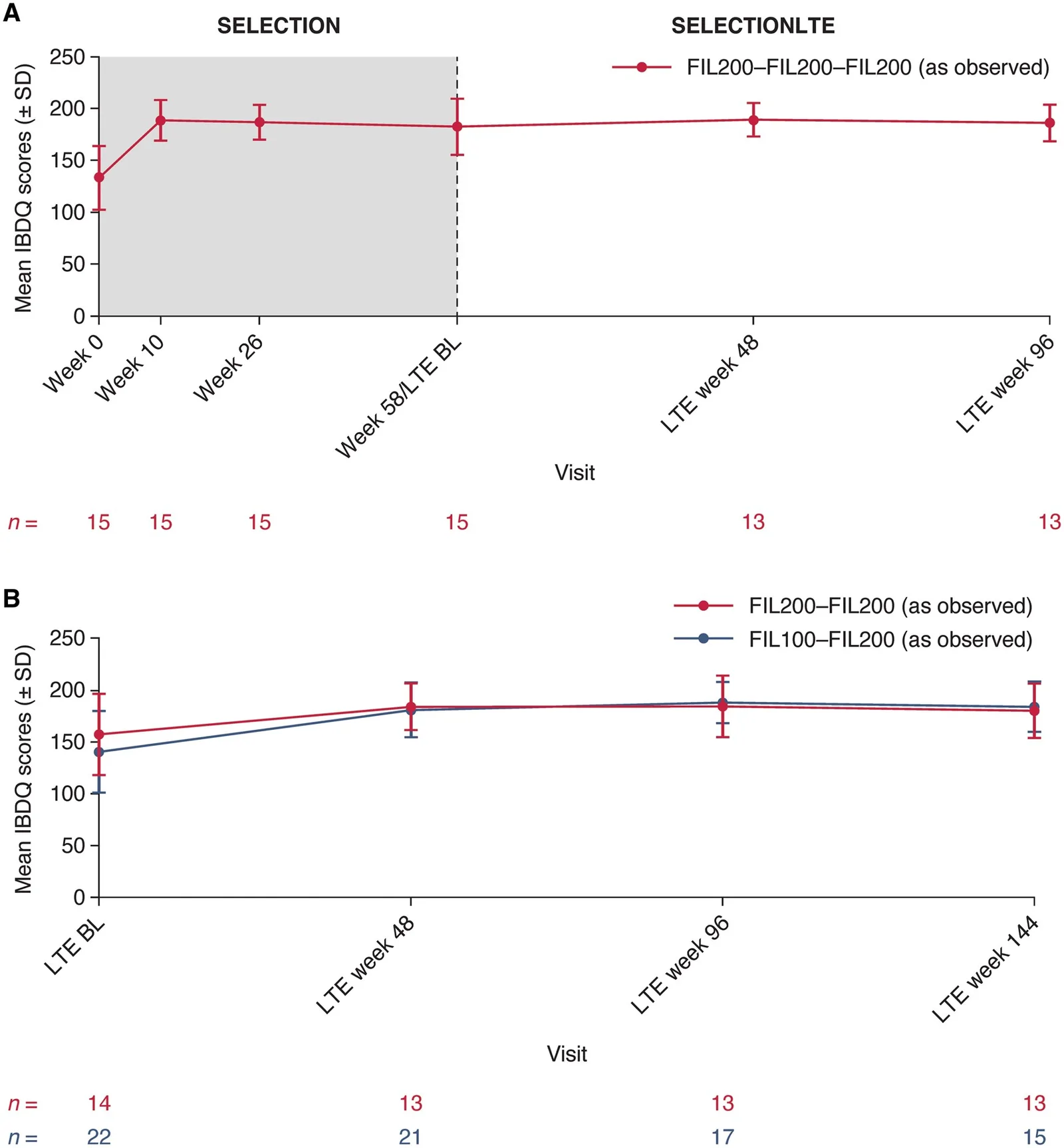
Mean IBDQ scores over time among Japanese (A) completers and (B) non-responders. The IBDQ is a 32-item questionnaire that produces a total score ranging from 32 to 224, with higher scores indicating better HRQoL.^23^ Abbreviations: BL, baseline; FIL100, filgotinib 100 mg; FIL200, filgotinib 200 mg; HRQoL, health-related quality of life; IBDQ, inflammatory bowel disease questionnaire; LTE, long-term extension; NRI, non-responder imputation; SD, standard deviation; SELECTIONLTE, SELECTION long-term extension.

The mean IBDQ score for FIL200–FIL200–FIL200 completers at LTE baseline was 182.7 (Figure 3A). The mean IBDQ score for these patients remained relatively constant in SELECTIONLTE, with a mean score of 186.3 observed at LTE Week 96 (Figure 3A). The mean IBDQ scores for FIL200–FIL200 and FIL100–FIL200 non–responders at LTE baseline were 157.0 and 140.1, respectively (Figure 3B). At LTE Week 144, the mean scores for FIL200–FIL200 and FIL100–FIL200 non–responders were 179.8 and 183.5, respectively (Figure 3B).

##### IBDQ remission in completers

**Figure 4:**
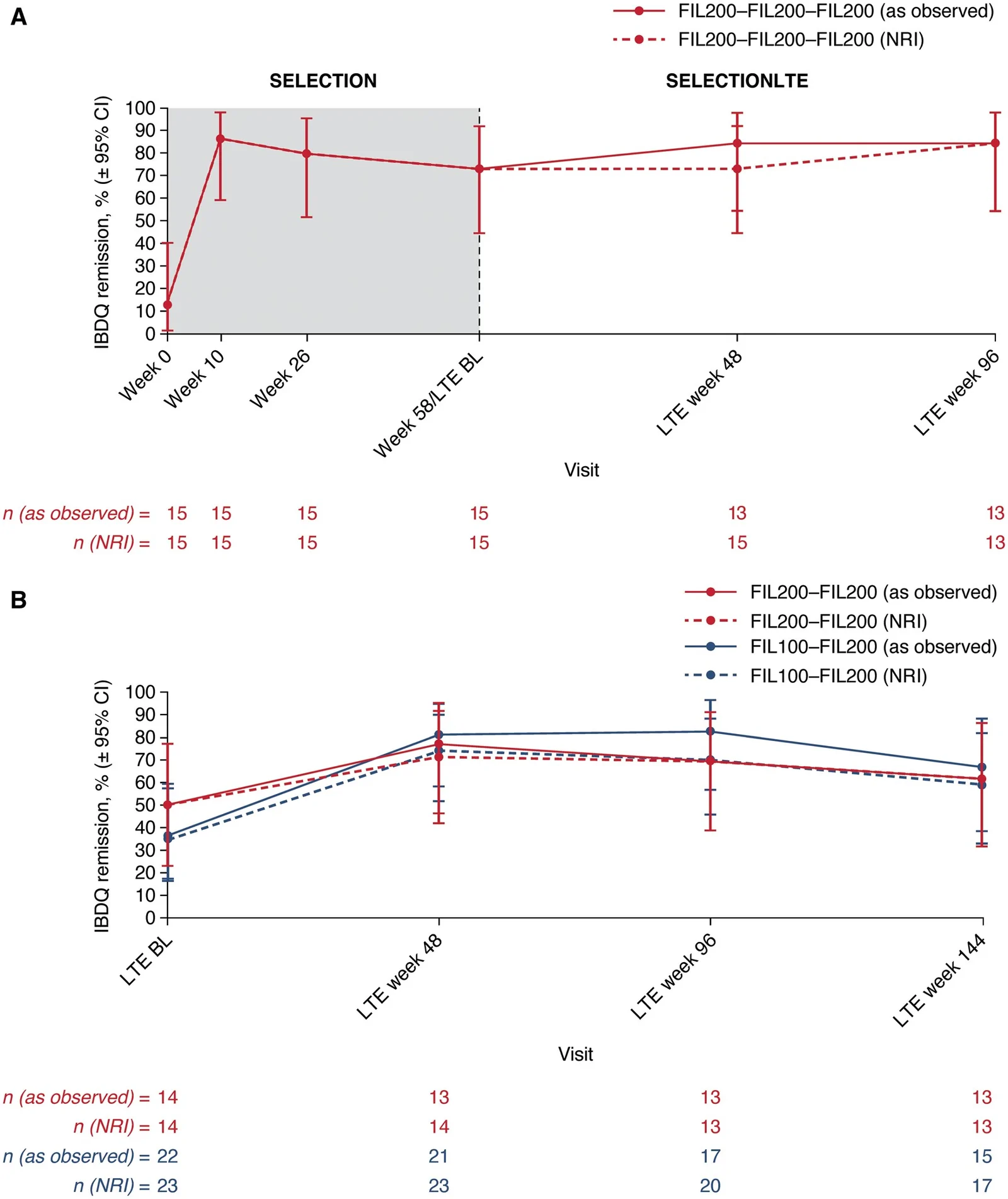
Proportions of Japanese (A) completers and (B) non-responders in IBDQ remission^a^ over time. The IBDQ is a 32-item questionnaire that produces a total score ranging from 32 to 224, with higher scores indicating better HRQoL.^23^ ^a^IBDQ remission was defined as an IBDQ score of ≥170. Abbreviations: BL, baseline; CI, confidence interval; FIL100, filgotinib 100 mg; FIL200, filgotinib 200 mg; IBDQ, inflammatory bowel disease questionnaire; LTE, long-term extension; NRI, non-responder imputation; SELECTIONLTE, SELECTION long-term extension.

In the as-observed analysis, the proportion of FIL200–FIL200–FIL200 completers who were in IBDQ remission at LTE baseline was 73.3% (11/15 patients) (Figure 4A). Rates in the LTE remained high, with 84.6% in remission at LTE Week 96 (11/13 patients) (Figure 4A). In the NRI analysis, similar trends were observed (Figure 4A).

##### IBDQ remission in non-responders

In the as-observed analysis, the proportions of FIL200–FIL200 and FIL100–FIL200 non–responders who were in IBDQ remission at LTE baseline were 50.0% (7/14 patients) and 36.4% (8/22 patients), respectively. At LTE Week 144, the rates for FIL200–FIL200 and FIL100–FIL200 non–responders in IBDQ remission increased to 61.5% (8/13 patients) and 66.7% (10/15 patients), respectively (Figure 4B). In the NRI analysis, similar trends were observed for the FIL200–FIL200 group (Figure 4B). For the FIL100–FIL200 group, the proportion of non–responders who were in IBDQ remission was similar at LTE baseline and was 58.8% at LTE Week 144, with the proportions generally lower than the as-observed data at time points between baseline and Week 144 (Figure 4B).

#### Fecal calprotectin

##### FCP remission in completers

**Figure 5:**
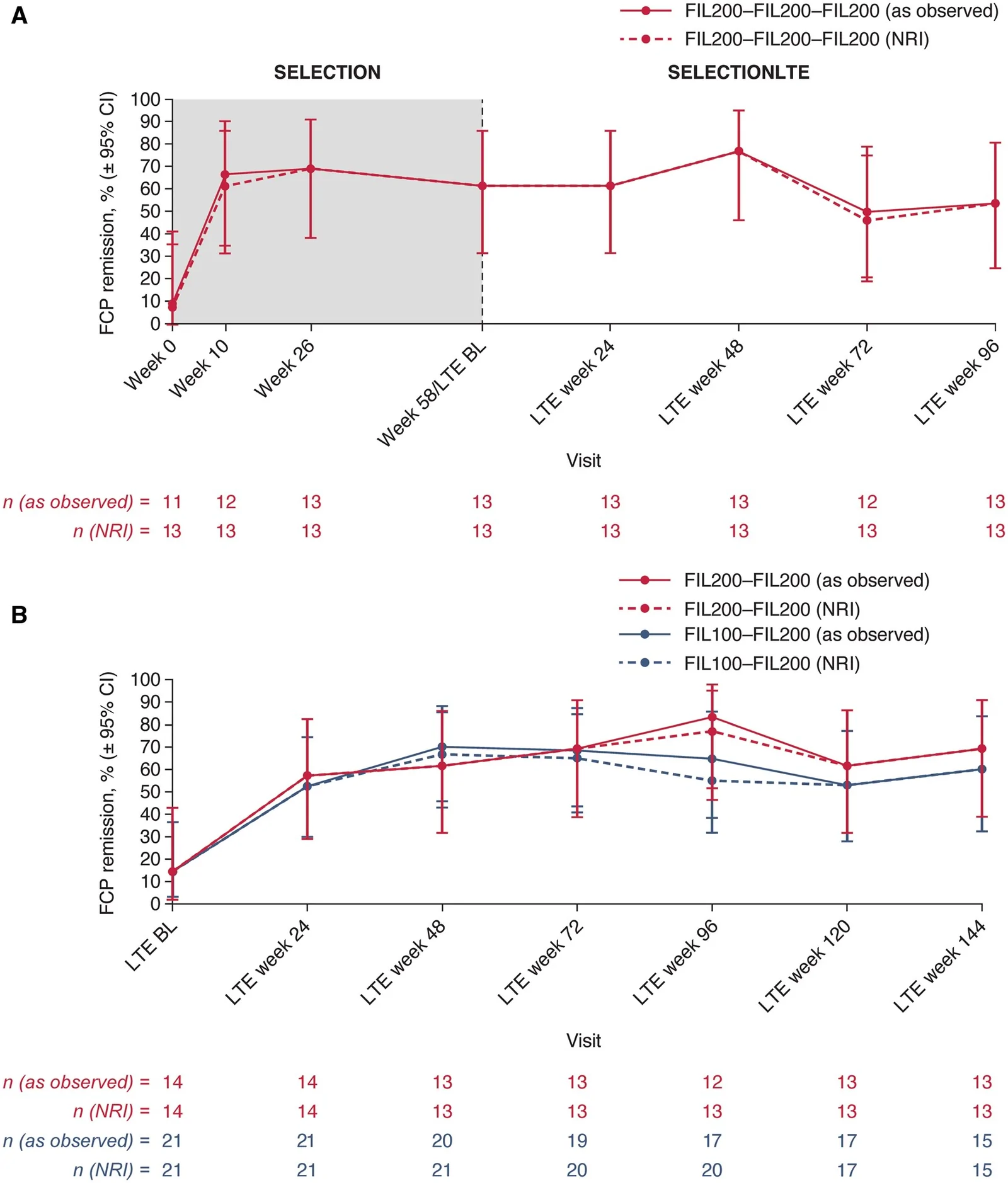
Proportions of Japanese (A) completers and (B) non-responders in FCP remission^a^ over time. ^a^FCP remission was defined as an FCP concentration of ≤250 μg/g. Abbreviations: BL, baseline; CI, confidence interval; FIL100, filgotinib 100 mg; FIL200, filgotinib 200 mg; FCP, fecal calprotectin; LTE, long-term extension; NRI, non-responder imputation; SELECTIONLTE, SELECTION long-term extension.

In the as-observed analysis, the proportion of FIL200–FIL200–FIL200 completers who were in FCP remission at LTE baseline was 61.5% (8/13 patients), and the rate was 53.8% at LTE Week 96 (7/13 patients) (Figure 5A). In the NRI analysis, similar trends were observed (Figure 5A).

##### FCP remission in non-responders

In the as-observed analysis, the proportions of FIL200–FIL200 and FIL100–FIL200 non–responders who were in FCP remission at LTE baseline were 14.3% (2/14 patients) and 14.3% (3/21 patients), respectively; the rates increased to 69.2% (9/13 patients) and 60.0% (9/15 patients), respectively, at LTE Week 144 (Figure 5B). In the NRI analysis, similar trends were observed (Figure 5B).

#### C-reactive protein

##### CRP levels in completers

For FIL200–FIL200–FIL200 completers, median CRP levels at LTE baseline and LTE Week 96 were 0.19 and 0.23 mg/L, respectively (Figure S2A).

##### CRP levels in non-responders

For FIL200–FIL200 and FIL100–FIL200 non–responders, median CRP levels at LTE baseline were 1.38 and 0.82 mg/L, respectively. Levels declined over time, reaching near-normal levels of 0.43 mg/L for FIL200–FIL200 non–responders and normal levels of 0.28 mg/L for FIL100–FIL200 non–responders by LTE Week 24. By LTE Week 144, median CRP levels for FIL200–FIL200 and FIL100–FIL200 non–responders further decreased to 0.22 and 0.20 mg/L, respectively (Figure S2B).

## Discussion

This interim analysis of the Japanese subpopulation in SELECTIONLTE, demonstrated the long-term safety profile and sustained efficacy of filgotinib in Japanese patients with moderately to severely active UC. FIL200 was effective in maintaining and improving long-term symptomatic remission and HRQoL, and in reducing inflammatory biomarker levels.

Ethnic differences can influence treatment efficacy and safety profiles. It is therefore meaningful in large multi–country studies to explore treatment outcomes in specific ethnic populations.^21^^,^^22^ The data reported in the present study are clinically relevant for understanding filgotinib efficacy and safety in Japanese patients and may offer useful insights for UC management in other Asian countries.

This interim analysis revealed a numerically higher incidence of overall infections and herpes zoster in Japanese patients than in the overall SELECTIONLTE population.^20^ Nasopharyngitis was the most common infection among Japanese patients and was also one of the most frequently reported TEAEs (grade 2 or higher) in the overall population.^20^ The EAIRs for overall infections and herpes zoster (including cutaneous disseminated cases) in Japanese patients who received FIL200 were 54.4 per 100 cPYE and 3.9 per 100 cPYE, respectively. In comparison, the rates in the overall FIL200 population were 35.5 per 100 cPYE and 1.5 per 100 cPYE, respectively.^20^ It is worth noting that all reported cases of herpes zoster in the Japanese population were mild or moderate in severity, and none were serious. In a previous *post hoc* analysis of Japanese patients from the SELECTION study, rates of herpes zoster in patients who received FIL200 were higher than those reported in the overall SELECTION population.^7^^,^^17^ Furthermore, in the SELECTIONLTE interim analysis of the overall population, it was noted that the EAIR for herpes zoster in patients from Asian countries was slightly higher than in other ethnicities.^20^ The EAIR for herpes zoster among Japanese patients has not been calculated in the integrated population of SELECTION and SELECTIONLTE studies. However, in the overall patients, the EAIR for herpes zoster in the integrated population was reported as 1.5 per 100 cPYE, which is almost comparable to EAIR of 1.4 per 100 cPYE in SELECTIONLTE. Based on these findings, it can be inferred that the EAIR for herpes zoster among Japanese patients in the integrated population is likely close to the EAIR of 3.9 per 100 cPYE observed in SELECTIONLTE in the present study.

These results contribute to existing data that suggest a higher risk of herpes zoster with JAK inhibitor use in patients from Asian countries compared with patients from Western countries.^21^^,^^24^^,^^25^ A meta-analysis of genome-wide association studies identified single nucleotide polymorphisms that may be associated with an increased risk of herpes zoster under JAK inhibitor treatment, including a variant near the *IL17RB* gene common in East Asian populations.^26^  *IL17RB* encodes a cytokine receptor that binds to interleukin 25 and interleukin 17 receptor B.^26^ This variant in *IL17RB* may cause an imbalance of T cell subtypes and a reduced threshold for varicella zoster virus reactivation, which is a potential mechanism for increased herpes zoster risk.^26^ In our study, a notable proportion (4/9; 44.4%) of the herpes zoster infections were reported within the first year of the LTE. However, cases were also reported in the 2nd, 3rd, and 4th years of the study, indicating that long-term vigilance for this type of AE is necessary, particularly in Asian populations. These results should, however, be interpreted with caution owing to the low number of patients in each group.

There were no occurrences of malignancy, MACEs, or VTEs in the Japanese subgroup. Among those who received FIL200 in the overall SELECTIONLTE population, the EAIRs in the interim analysis were 0.5 per 100 cPYE for malignancies (excluding NMSC), 0.6 per 100 cPYE for NMSC, 0.2 per 100 cPYE for MACEs, and 0.0 per 100 cPYE for VTEs.^20^ The absence of these TEAEs of interest in the Japanese subpopulation is likely due to the small sample size.

In the current study, approximately 47% of completers and 40% of non–responders were receiving systemic CS therapy at SELECTION induction baseline. Although the small sample size in our study limits the evaluation of the impact of concomitant CS use in the Japanese population, neither concomitant CS use nor IM use was identified as a predictor of clinical remission in Japanese real-world data.^27^ Furthermore, a previous *post hoc* analysis of SELECTION data demonstrated that the safety and efficacy profiles of FIL200 were comparable regardless of concomitant IM use at induction baseline.^28^

Continued filgotinib therapy in SELECTIONLTE was efficacious in maintaining and improving symptomatic remission and HRQoL in Japanese patients. For completers, improvements in mean pMCS, pMCS remission, and HRQoL observed in SELECTION were generally maintained in SELECTIONLTE up to LTE Week 96. In non–responders, improvements in mean pMCS, and increases in the proportions of patients achieving pMCS remission, were also observed in SELECTIONLTE up to LTE Week 144. These results suggest that gradual improvements are observed over time in patients who do not initially respond to filgotinib treatment. Among non–responders, both FCP remission rates and CRP levels improved during the LTE.

The main strength of this study is the long-term follow-up, which provides insights into the long-term benefits and risks associated with FIL200 use in Japanese patients. Limitations of this study include those inherent to an LTE, such as its open-label nature, the lack of a comparator, and the small sample size. This was also an interim analysis with a staggered entry of patients from the parent study (ie, not all patients had reached the later time points before the data cut-off), which resulted in very small sample sizes at later time points. Finally, the outcomes of the NRI analyses should be interpreted with caution, as the number of patients included in the analyses changed over time due to patient discontinuation.

## Conclusion

In summary, findings of this study show that filgotinib treatment was efficacious in maintaining and improving symptomatic remission and HRQoL, and in reducing inflammatory biomarker levels over a maximum of 3 years, resulting in an acceptable long-term benefit–risk profile for Japanese patients. Numerically higher incidences of overall infections and herpes zoster infection were observed in Japanese patients in SELECTIONLTE compared with the overall SELECTIONLTE population. SELECTIONLTE is ongoing and the final analysis of safety and efficacy over the 336-week study period will be reported upon completion.

## Supplementary Material

otag006_Supplementary_Data

## Data Availability

Gilead Sciences shares anonymized individual patient data upon request, or as required by law or regulation, with qualified external researchers based on submitted curriculum vitae and reflecting non–conflict of interest. The request proposal must also include a statistician. Approval of such requests is at Gilead Science’s discretion and is dependent on the nature of the request, the merit of the research proposed, the availability of the data, and the intended use of the data. Data requests should be sent to datarequest@gilead.com.
