# Supplementary material for: Safety and efficacy of long-term filgotinib treatment in Japanese patients with ulcerative colitis: an interim subgroup analysis of the SELECTION long-term extension study
Source: Crohns Colitis 360. 2026 Jan 28;8(1):otag006. doi: 10.1093/crocol/otag006 (PMC12924877; doi:10.1093/crocol/otag006)
Supplement: otag006_Supplementary_Data [file otag006_supplementary_data.zip › SELECTIONLTE_Japanese_manuscript_Supplementary_Data_10Feb2026.docx]

**SUPPLEMENTARY MATERIALS
Plain language summary**

Ulcerative colitis (UC) is a long-term disease where the large intestine is inflamed. Patients with UC have symptoms such as blood in stool, diarrhea, urgent bowel movements, tiredness, and abdominal pain. In Japan, the number of people diagnosed with UC has increased over time.

Filgotinib is an oral drug that was approved in Japan in 2022 for treating patients with UC after a clinical study called SELECTION. SELECTION showed that filgotinib once daily at a dose of 200 mg was well tolerated and controlled UC intestinal symptoms, levels of inflammatory biomarkers in blood and stool, and improved health-related quality of life over 58 weeks. Researchers are now looking into the long-term effects of daily filgotinib treatment in a study called SELECTION long-term extension (SELECTIONLTE). Here, we present safety and efficacy results that were collected over almost 4 years and 3 years, respectively, for Japanese patients participating in SELECTIONLTE.

In this study, completers were Japanese patients who completed the 58-week SELECTION study because they showed a good response to filgotinib 200 mg treatment at week 10. Completers continued receiving this dose in SELECTIONLTE. In contrast, Japanese patients who did not show a good response to filgotinib 100 mg or 200 mg treatment at week 10 in SELECTION were referred to as non-responders. Non-responders entered the SELECTIONLTE study at week 11 without completing SELECTION and were given the maximum dose of filgotinib (200 mg).

This study did not find any new safety concerns for the long-term treatment of Japanese patients with filgotinib 200 mg. However, Japanese patients had a slightly higher risk of infections, including herpes zoster (also known as shingles), compared with the overall group of patients in SELECTIONLTE. The cases of herpes zoster were generally mild in severity. The study also found that filgotinib 200 mg continued to control and improve UC intestinal symptoms, levels of inflammatory biomarkers in blood and stool, and health-related quality of life in Japanese patients over the 3-year period. Furthermore, Japanese patients who did not show a good response to their filgotinib treatment at week 10 in SELECTION (non-responders) benefited from long-term treatment with filgotinib 200 mg in SELECTIONLTE. As SELECTIONLTE is ongoing, the final results will be published once the study is complete.

The results of this study suggest that, for Japanese patients, filgotinib is an effective long-term option for managing UC, although Japanese patients seem to have a higher risk of herpes zoster infection compared with the overall SELECTIONLTE population.

**Table S1** TEAEs of infections and infestations among Japanese patients who had received at least one dose of FIL200.

|  | **FIL200**  **(cPYE = 240.6)**  **(*n* = 86)** | |
| --- | --- | --- |
|  | *n*/cPYE | EAIR per 100 cPYE (95% CI) |
| **Infections and infestations** | 60/110.2 | 54.4 (41.5–70.1) |
| Nasopharyngitis | 29/163.5 | 17.7 (11.9–25.5) |
| Herpes zoster | 8/228.1 | 3.5 (1.5–6.9) |
| Influenza | 8/220.9 | 3.6 (1.6–7.1) |
| Pharyngitis | 6/228.1 | 2.6 (1.0–5.7) |
| Gastroenteritis | 5/230.1 | 2.2 (0.7–5.1) |
| Bronchitis | 4/233.4 | 1.7 (0.5–4.4) |
| COVID-19 | 4/239.6 | 1.7 (0.5–4.3) |
| Hordeolum | 4/237.2 | 1.7 (0.5–4.3) |
| Conjunctivitis | 3/232.1 | 1.3 (0.3–3.8) |
| Gingivitis | 2/237.6 | 1.3 (0.3–3.7) |
| Oral herpes | 3/232.9 | 1.3 (0.3–3.8) |
| Upper respiratory tract infection | 3/235.4 | 1.3 (0.3–3.7) |
| Abscess limb | 2/235.3 | 0.8 (0.1–3.1) |
| Anal abscess | 2/239.6 | 0.8 (0.1–3.0) |
| Helicobacter infection | 2/236.3 | 0.8 (0.1–3.1) |
| Tonsillitis | 2/239.0 | 0.8 (0.1–3.0) |
| Urinary tract infection | 2/236.9 | 0.8 (0.1–3.0) |
| Acute sinusitis | 1/238.3 | 0.4 (0.0–2.3) |
| Campylobacter gastroenteritis | 1/238.5 | 0.4 (0.0–2.3) |
| Clostridium difficile colitis | 1/240.5 | 0.4 (0.0–2.3) |
| Clostridium difficile infection | 1/238.5 | 0.4 (0.0–2.3) |
| Cystitis | 1/238.8 | 0.4 (0.0–2.3) |
| Cytomegalovirus enteritis | 1/240.5 | 0.4 (0.0–2.3) |
| Dermatophytosis of nail | 1/239.4 | 0.4 (0.0–2.3) |
| Diverticulitis | 1/240.5 | 0.4 (0.0–2.3) |
| Enteritis infectious | 1/239.7 | 0.4 (0.0–2.3) |
| Enterocolitis infectious | 1/240.0 | 0.4 (0.0–2.3) |
| Furuncle | 1/237.3 | 0.4 (0.0–2.3) |
| Gastroenteritis viral | 1/238.0 | 0.4 (0.0–2.3) |
| Helicobacter gastritis | 1/238.0 | 0.4 (0.0–2.3) |
| Herpes zoster cutaneous disseminated | 1/240.5 | 0.4 (0.0–2.3) |
| Impetigo | 1/240.1 | 0.4 (0.0–2.3) |
| Nasal herpes | 1/240.1 | 0.4 (0.0–2.3) |
| Periodontitis | 1/240.0 | 0.4 (0.0–2.3) |
| Pneumonia aspiration | 1/240.5 | 0.4 (0.0–2.3) |
| Respiratory tract infection | 1/237.9 | 0.4 (0.0–2.3) |
| Viral pharyngitis | 1/238.4 | 0.4 (0.0–2.3) |

CI, confidence interval; cPYE, censored patient-years of exposure; EAIR, exposure-adjusted incidence rate; FIL200, filgotinib 200 mg; COVID-19, coronavirus disease 2019; TEAE, treatment-emergent adverse event.

**Table S2** Narratives for TEAEs related to herpes zoster infections.

| **TEAE (reported term)** | **Day of onset^a^** | **Age, (years), sex** | **Toxicity grade^b^** |
| --- | --- | --- | --- |
| Herpes zoster | 30 | 36, M | 1 |
| Herpes zoster | 151 | 68, M | 2 |
| Herpes zoster | 222 | 28, M | 2 |
| Herpes zoster | 353 | 57, M | 2 |
| Herpes zoster | 404 | 22, M | 1 |
| Herpes zoster cutaneous disseminated | 867 | 54, M | 2 |
| Herpes zoster | 886 | 56, M | 2 |
| Herpes zoster | 1191 | 36, M | 2 |
| Herpes zoster | 1235 | 50, F | 2 |

Note: no herpes zoster infection was classified as serious.

^a^Day of onset = AE start date − treatment start date + 1 day.

^b^Toxicity grades: 1, mild; 2, moderate; 3, severe; 4, life-threatening.

AE, adverse event; F, female; M, male; TEAE, treatment-emergent adverse event.


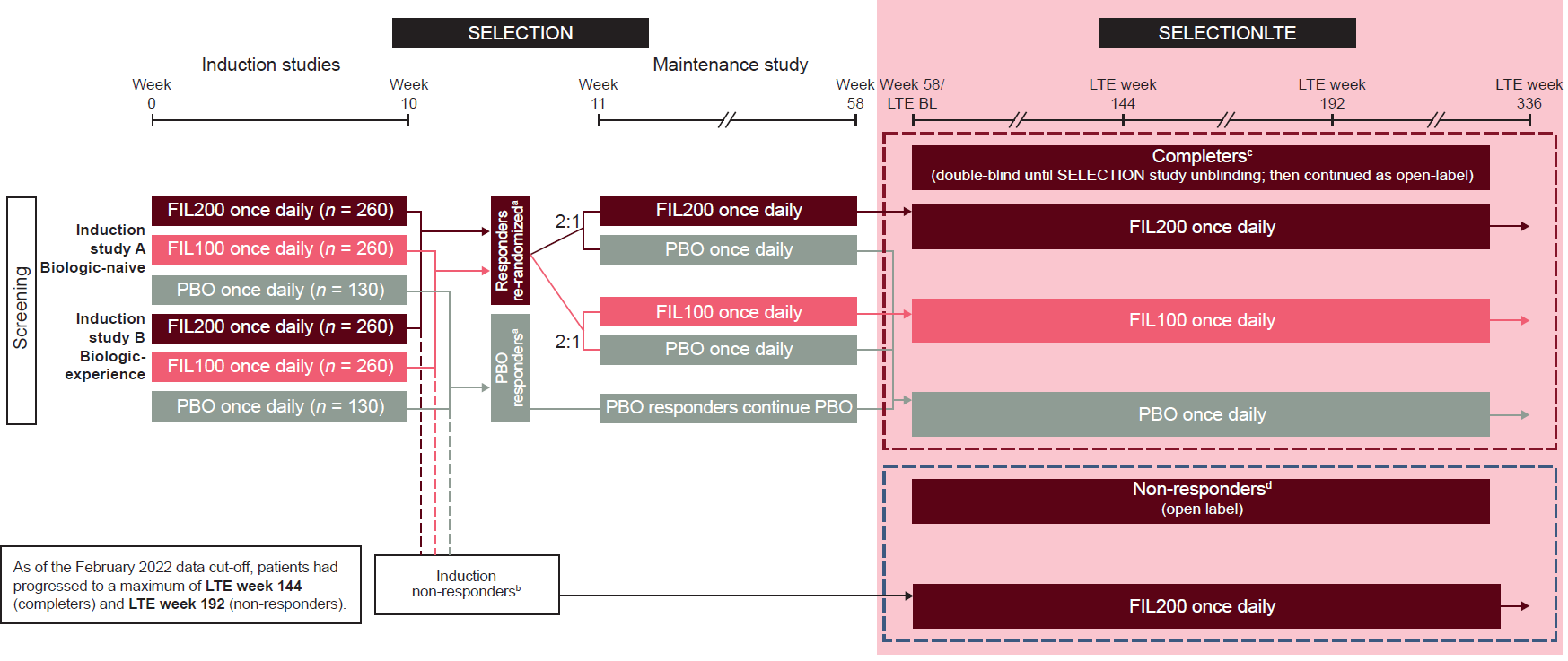


**Figure S1** Study designs of the SELECTION and SELECTIONLTE trials.

^a^Responders were defined as patients in clinical remission (defined as a Mayo endoscopic subscore of 0 or 1, a rectal bleeding subscore of 0, and a stool frequency subscore of 0 or 1 [with a decrease of at least 1 point from induction baseline]) or with an MCS response (defined as a decrease in MCS of at least 3 points and 30% or more from baseline, with a decrease in rectal bleeding subscore of 1 point or more, or an absolute rectal bleeding subscore of 0 or 1) at SELECTION week 10. MCS was defined as the sum of endoscopic, rectal bleeding, stool frequency, and Physician's Global Assessment subscores, with the total score ranging from 0 to 12.

^b^Induction non-responders and patients who experienced protocol-specified disease worsening during the maintenance phase of SELECTION (defined as an increase in pMCS of ≥ 3 points from the week 10 value on two consecutive visits to achieve a score ≥ 5 or an increase in pMCS on two consecutive visits to achieve a score of 9 if the week 10 value was > 6).

^c^Completers were defined as induction responders who completed SELECTION to week 58; in SELECTIONLTE, they continued receiving the same treatment as in SELECTION.

^d^Non-responders were induction non-responders. In SELECTIONLTE, non-responders received the maximum dose of open-label filgotinib, as permitted in the country in which they resided (FIL200 for Japanese patients).

BL, baseline; FIL100, filgotinib 100 mg; FIL200, filgotinib 200 mg; LTE, long-term extension; MCS, Mayo Clinic Score; pMCS, partial Mayo Clinic Score.
Reproduced from Aliment Pharmacol Ther, “Long-term safety and efficacy of filgotinib for the treatment of moderately to severely active ulcerative colitis: Interim analysis from up to 4 years of follow-up in the SELECTION open-label long-term extension study” by Feagan BG, Matsuoka K, Rogler G, Laharie D, Vermeire S, Danese S, et al. © 2024 John Wiley & Sons Ltd. Reproduced with permission of John Wiley & Sons Ltd.


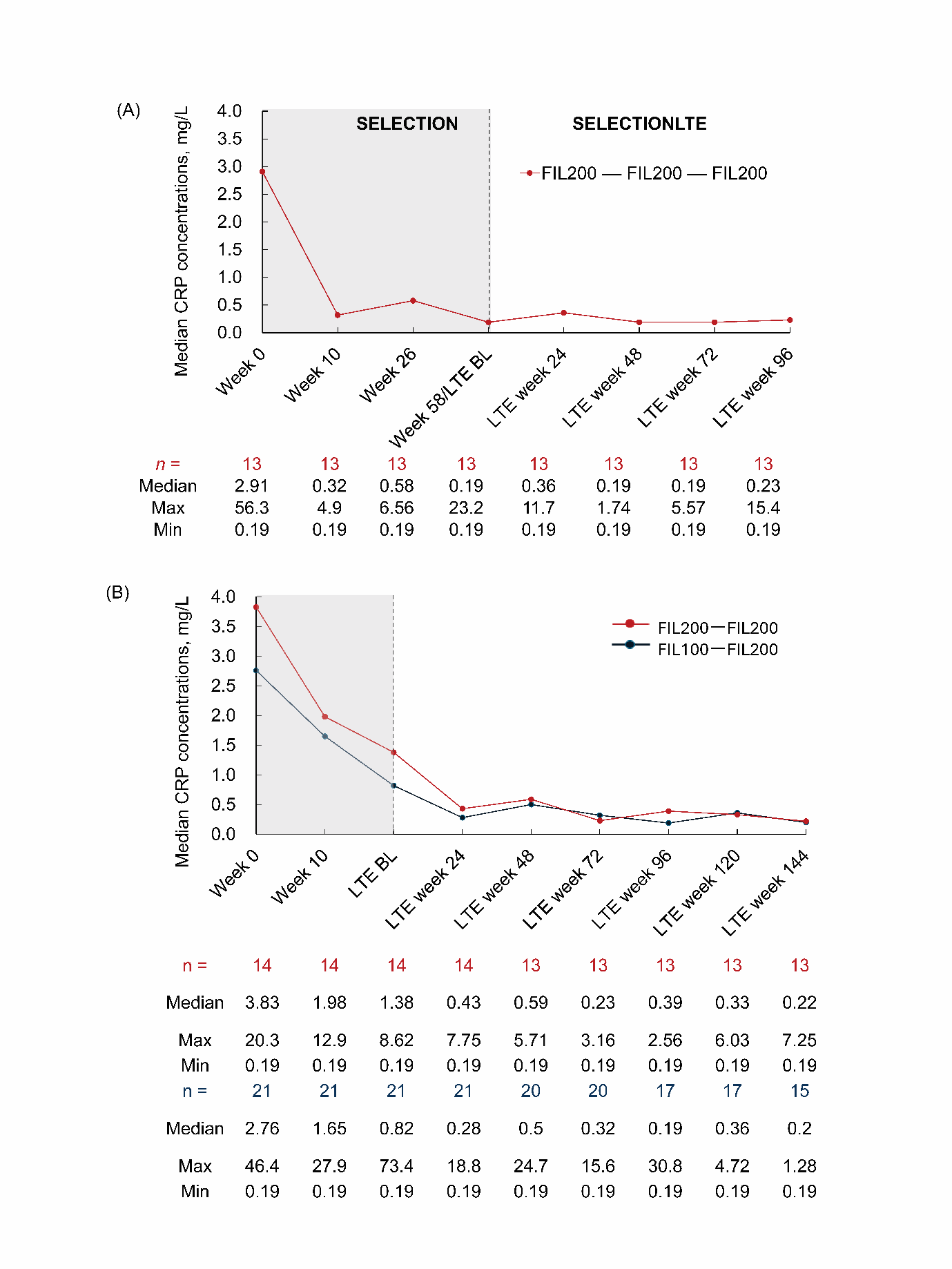


**Figure S2** Median CRP concentrations over time among Japanese A) completers and B) non-responders.

BL, baseline; CRP, C-reactive protein; FIL100, filgotinib 100 mg; FIL200, filgotinib 200 mg; LTE, long-term extension, Max, maximum; Min, minimum; SELECTIONLTE, SELECTION long-term extension.
